# Lutein Protects RGC-5 Cells Against Hypoxia and Oxidative Stress

**DOI:** 10.3390/ijms11052109

**Published:** 2010-05-11

**Authors:** Suk-Yee Li, Amy C. Y. Lo

**Affiliations:** 1 Eye Institute, Li Ka Shing Faculty of Medicine, The University of Hong Kong, Pokfulam, Hong Kong; E-Mail: sukyeeli@hku.hk; 2 Research Centre of Heart, Brain, Hormone and Healthy Aging, The University of Hong Kong, Pokfulam, Hong Kong

**Keywords:** antioxidants, carotenoids, cobalt chloride, hydrogen peroxide, ischemia, RGC-5

## Abstract

Retinal ischemia and oxidative stress lead to neuronal death in many ocular pathologies. Recently, we found that lutein, an oxy-carotenoid, protected the inner retina from ischemia/reperfusion injury. However, it is uncertain whether lutein directly protects retinal ganglion cells (RGCs). Here, an *in vitro* model of hypoxia and oxidative stress was used to further investigate the neuroprotective role of lutein in RGCs. Cobalt chloride (CoCl_2_) and hydrogen peroxide (H_2_O_2_) were added to a transformed RGC cell line, RGC-5, to induce chemical hypoxia and oxidative stress, respectively. Either lutein or vehicle was added to cultured cells. A higher cell count was observed in the lutein-treated cells compared with the vehicle-treated cells. Our data from this *in vitro* model revealed that lutein might protect RGC-5 cells from damage when exposed to either CoCl_2_-induced chemical hypoxia or H_2_O_2_-induced oxidative stress. These results suggest that lutein may play a role as a neuroprotectant.

## 1. Introduction

Retinal ischemia leads to irreversible neuronal injury and visual impairment in many ocular pathologies such as glaucoma, diabetic retinopathy (DR) and retinal vessel occlusion [1]. Retinal ganglion cell (RGC) death is common in these ocular pathologies. During ischemia, depletion of ATP stores, ions imbalance, glutamate excitotoxicity, apoptosis and free radical production eventually lead to RGC death [1–3]. Reperfusion following ischemia results in oxidative stress, which also plays a role in RGC damage [4,5]. Investigations have been carried out to study the neuroprotection of RGCs using carotenoids [6].

Lutein ((3*R*,39*R*,69*R*)-b,e-carotene-3,39-diol) is a member of xanthophyll dietary carotenoids and structurally similar to zeaxanthin [7,8]. These xanthophylls have a chemical formula of C_40_H_56_O_2_ with a hydroxyl group attached to each end of the molecule. The difference between lutein and zeaxanthin is the position of a double bond in one of the hydroxyl groups [8]. The unique structure enables lutein to react more strongly with singlet oxygen than other carotenoids [9]. Like zeaxanthin, lutein is predominately present in the macular region and acts as an efficient pigment for absorbing high energy blue light and a direct free radical scavenger to prevent macular damage [8,10]. However, lutein cannot be synthesized in the body and need to be obtained in the diet. It is richly found in dark green leafy vegetables and eggs [11].

Recently, our group demonstrated that lutein protected the inner retina from damage after ischemia/reperfusion *in vivo* [12]. We showed that lutein was anti-apoptotic and prevented cell damage by decreasing oxidative stress. However, the effect of lutein on specific cell populations is unknown. In this study, a transformed cell line of RGC, RGC-5, was used. This cell line was originally derived by transforming postnatal day one rat retinal cells with Ψ_2_ E1A virus [13]. RGC-5 cells express RGC-specific markers such as Brn-3c and Thy-1 although they are mitotically active which is different from RGCs. Here, we sought to investigate whether lutein could reverse the cytotoxic effect of hypoxia or oxidative stress, key events during ischemic injury, specifically on RGC-5 *in vitro*.

## 2. Results and Discussion

### 2.1. Results

**Figure 1.**
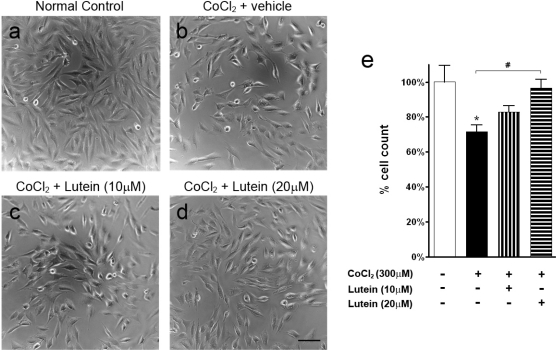
Light micrographs and cell count of RGC-5 cells treated with cobalt (II) chloride (CoCl_2_; 300 μM). (**a**) Normal control. (**b**) Vehicle treatment. (**c**) Lutein treatment at 10 μM. (**d**) Lutein treatment at 20 μM. CoCl_2_-induced hypoxia led to cell death in the vehicle-treated group (b) compared with control (a). However, 20 μM lutein treatment reversed the cytotoxic effect of CoCl_2_ (d). (**e**) Count of RGC-5 cells treated with CoCl_2_ referenced to the normal control. A decreased cell number was observed for the vehicle-treated group (*p < 0.05 *versus* control). However, an increased RGC-5 cell number was observed after 20μM lutein treatment (^#^p < 0.05 *versus* vehicle-treated). Scale bar, 25 μm. Error bars, SEM.

Chemical hypoxia was induced in RGC-5 cells using cobalt (II) chloride (CoCl_2_). After hypoxia, profound cell loss was observed in the vehicle-treated hypoxic group (Figure 1b,e; p < 0.05 *versus* normal control). Cells appeared to be more round, with loss of processes (Figure 1b) when compared with the normal control (Figure 1a). However, lutein treatment reversed the cytotoxic effect of CoCl_2_ and led to less damage to RGC-5 cells (Figure 1c,d). Cells treated with 20 μM lutein (Figure 1d) showed morphology similar to that of the normal control (Figure 1a). Quantitative analysis by cell counting showed that more RGC-5 cells were observed in the lutein-treated group (Figure 1e; p < 0.05 at 20 μM *versus* vehicle-treated group).

**Figure 2.**
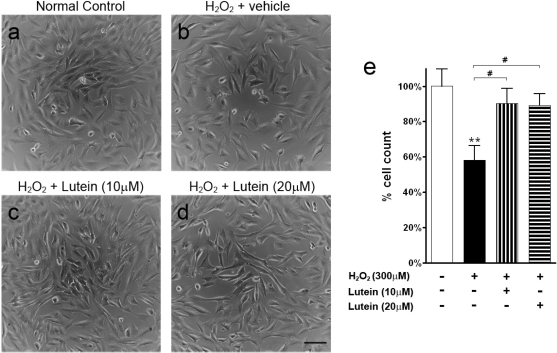
Light micrographs and cell count of RGC-5 cells treated with hydrogen peroxide (H_2_O_2_; 300 μM). (**a**) Normal control. (**b**) Vehicle treatment. (**c**) Lutein treatment at 10 μM. (**d**) Lutein treatment at 20 μM. H_2_O_2_-induced oxidative stress led to cell death in the vehicle-treated group (b). Lutein treatment reversed the cytotoxic effect (c and d). (**e**) Cell count in RGC-5 cells treated with H_2_O_2_. Cell count referenced to the normal control. H_2_O_2_ exposure led to a decrease in cell number in the vehicle-treated group (**p < 0.01 *versus* control). However, both 10 μM and 20 μM lutein treatment protected RGC-5 cells from damage (^#^p < 0.05 *versus* vehicle-treated control). Scale bar, 25 μM. Error bars, SEM.

Hydrogen peroxide (H_2_O_2_) was used to induce oxidative stress. Exposure to H_2_O_2_ led to cell death in the vehicle-treated group (Figure 2b,e; p < 0.01 *versus* normal control). Upon lutein treatment at both at 10 μM and 20 μM, the number of RGC-5 cells was increased (Figure 2c,d,e; p < 0.05 *versus* vehicle-treated group) to a number similar to that of the normal control (Figure 2e; p > 0.05).

### 2.2. Discussion

Ischemia and oxidative stress are common causes of many ocular diseases, which lead to irreversible RGC damage. In the present study, we examined the neuroprotective effect of lutein on RGC-5 cells against CoCl_2_-induced chemical hypoxia and H_2_O_2_-induced oxidative stress *in vitro*. Our data demonstrated that lutein exerted neuroprotection on RGC-5 cells against hypoxia and oxidative stress.

Retinal ischemia is a feature of many ocular pathologies such as glaucoma, DR and retinal vessel occlusion [1]. In experimental studies, CoCl_2_ is one of the common agents to induce hypoxia [14–18]. CoCl_2_ treatment simulates hypoxia, a key event during ischemic injury, by altering gene and protein expression similarly to ischemia [19]. It induces hypoxia by blocking the degradation of hypoxia-inducible factor-1α (HIF-1α) and subsequent HIF-1α accumulation [20]. Moreover, CoCl_2_ also induced apoptosis through activation of caspase-3/8, cleavage of anti-apoptotic protein Mcl-1 and generation of reactive oxygen species (ROS) in a variety of *in vitro* studies [17,21]. In animal studies, CoCl_2_ has been shown to induce apoptosis and retinal photoreceptor degeneration [22]. In addition, CoCl_2_-induced hypoxia has been adopted in RGC *in vitro* [16,23] and *in vivo* [16]. Accumulation of HIF-1α protein increased expression of heat shock protein-27, and generation of β-amyloid peptide [23] was shown in CoCl_2_-treated RGC-5 cells. In our results, we demonstrated that CoCl_2_ attributed to hypoxia-induced injury in RGC-5 cells.

Oxidative stress is one of the key factors leading to neuronal injury. The retina is highly susceptible to oxidative stress because of the high content of polyunsaturated fatty acids and high oxygen consumption [24]. Under normal situations, cells possess several intrinsic antioxidant enzymes such as superoxide dismutase, catalase and glutathione peroxidase to cope with oxidative stress resulted from normal metabolism in our body [3]. However, during injuries such as in ischemia/reperfusion, glaucoma and DR, overproduction of ROS and free radicals overwhelms the intrinsic antioxidant mechanisms [1,3]. RGC is sensitive to oxidative stress in pathological situations *in vivo* [3] and *in vitro* [25]. In experimental studies, H_2_O_2_ is widely used to induce oxidative stress [25]. Exogenous H_2_O_2_ increases intracellular accumulation of ROS [26], apoptosis and leads to loss of cell viability [25] in RGC-5. H_2_O_2_-induced apoptosis in RGCs has been shown to be caspase-independent and yet involves the activation of poly(ADP-ribose) polymerase and apoptosis-inducing factor [25]. In the present study, H_2_O_2_ also induced significant cell damage to RGC-5 cells, which was comparable to previously found [25].

In the macula, lutein absorbs high energy blue light and protects the retina from oxidative injury [10]. Low levels of lutein intake have been shown to associate with the prevalence of AMD [27]. Lutein supplementation has been shown to improve vision and retard progression of AMD in clinical trial studies [28,29]. To our knowledge, there is no reported toxic effect of lutein even at a high dose of intake [30]. No significant clinical, hematological, biochemical or histopathological side effects were noted in rats fed with 733 mg/kg per day of purified crystalline lutein [10,30]. More importantly, lutein has been regarded and approved safe to be used as a daily supplement and to be included into certain food and beverage application in USA [10]. However, the use of lutein is still limited in treating AMD, which is an outer retinal disease.

Recently, intensive efforts have been made to explicate the neuroprotective effects of carotenoids in ocular diseases *in vivo* [12,31,32] and *in vitro* [5,33]. Lutein treatment in DR mice restored malondialdehyde and glutathione protein levels, glutathione peroxidase activity as well as electroretinogram response to control values [31]. Lutein also reversed the activation of factor-kappa B transcription, which is involved in oxidative stress and inflammation response [31]. In mice with retinal inflammation, lutein reduced inflammatory response and oxidative stress through reversal of STAT3 activation, downstream of inflammatory cytokine signals [32]. In addition, the activation of glial fibrillary acidic protein, an indicator of pathological change of Muller glial cells, was prevented in animal treated with lutein. In our recently reported study, we found that lutein was also protective to inner retinal neurons in ischemia/reperfusion injury *in vivo* [12]. Reduced immunoreactivity of nitrotyrosine and poly(ADP-ribose) in the inner retina, indicating a reduced oxidative stress, was observed in lutein-treated ischemic retina. Effects of carotenoids on a specific cell population were investigated using an *in vitro* approach. Zeaxanthin and astaxanthin have been shown to protect RGC-5 cells from oxidative injuries [5,33]. In the present study, our results suggested that lutein treatment protected RGC-5 from CoCl_2_-induced chemical hypoxia and H_2_O_2_-induced oxidative stress. Indeed, it has been proposed that lutein can effectively reduce the intracellular accumulation of H_2_O_2_ by scavenging H_2_O_2_ and superoxide as well as inhibit NFκB-regulated inflammatory gene expression in lipopolysaccharide-stimulated macrophages *in vivo* and *in vitro* [34]. This indicates that lutein is able to penetrate into cells and scavenge intracellular H_2_O_2_ to prevent cell damage. Furthermore, lutein-binding protein [35] and retinal tubulin [36] are found in the ganglion cell layer of primate retina and bovine retina. These proteins are suggested to be involved in lutein transport. However, further study is necessary to investigate the expression and localization of lutein-bind protein in rodents and the RGC-5 cell line.

The RGC-5 cell line was previously used as a RGC-specific *in vitro* model [4,5,18,26,33]. However, several recent reports have questioned the validity of this cell line. It was demonstrated that RGC-5 cells lack critical biochemical and physiological RGC properties [37–39]. In addition, RGC-5 cells do not express RGC-specific markers such as neurofilaments or Thy 1.2 [38]. Moreover, RGC-5 cells are unexcitable, with no voltage-dependent inward Na^+^ or Ca^2+^ currents or action potentials, which are critical properties of cultured postnatal and adult rat RGCs [37]. All these pieces of evidence imply the limitation of using the RGC-5 cell line as an *in vitro* model of RGCs. Primary RGC culture may be a more appropriate *in vitro* model of RGC study.

## 3. Experimental Section

RGC-5 cells (ATCC, VA, USA) were routinely maintained in Dulbecco’s modified Eagle’s medium (DMEM; Gibco, Carlsbad, CA, USA) supplemented with 10% fetal bovine serum (FBS; Gibco), 100 U/mL penicillin and 100 μg/mL streptomycin (Gibco). Cells were grown in a humidified incubator of 95% air and 5% CO_2_ at 37 °C. Cells were passaged when 80% confluent.

For cell counting studies, RGC-5 cells were seeded in 96-well plates at a density of 5,000 cells/well in DMEM with 10% FBS for 24 hours. Hypoxia and oxidative stress was induced by incubating the cells with CoCl_2_ (300 μM; Sigma-Aldrich, St. Louis, MO, USA) and H_2_O_2_ (300 μM; BDH Chemicals Ltd., Atherstone, UK) in DMEM with 1% FBS for 24 hours. Either Lutein (10 μM and 20 μM; Sigma) or vehicle (0.01% dimethyl sulfoxide (DMSO); Sigma) was added to the culture medium at the onset of injury. The concentrations of CoCl_2_, H_2_O_2,_ and lutein used were adopted from previous studies [4,14,18,34,40]. Photographs from each well of the culture plates were captured under light microscope (Eclipse TE2000-5; Nikon, Tokyo, Japan) using a digital camera (Spot Flex; Diagnostic Instruments, Inc., Sterling Heights, MI, USA). Five fields (300 μm × 300 μm) were selected from each photograph for cell counting. The experiments were performed in duplicate and repeated four times.

Quantitative results were expressed as mean ± SEM. One-way ANOVA tests, followed by Bonferroni’s multiple comparison tests, were used to test the statistical significance of differences among the groups. Significance was set at p < 0.05.

## 4. Conclusions

In the present study, we demonstrated that lutein can protect RGC-5 cells from injury induced by CoCl_2_-induced chemical hypoxia or H_2_O_2_-induced hypoxia or oxidative stress. These results suggest that lutein may play a role as a neuroprotectant.
